# Supplementary material for: Identification of cell-type specific alternative transcripts in the multicellular alga Volvox carteri
Source: BMC Genomics. 2023 Oct 30;24:654. doi: 10.1186/s12864-023-09558-0 (PMC10617192; doi:10.1186/s12864-023-09558-0)
Supplement: Supplementary file 1 — Additional file 1: Figure S1. Verification gene and transcript expression within replicates. Figure S2. Verification of differences in gene and transcript expression between cell types. Figure S3. Alternate restriction criteria: all genes with two transcripts. Figure S4. Alternate restriction criteria: two oppositely expressed transcripts with large biases. Figure S5. Hand Curation Examples. Figure S6. Convergent Transcription Examples. Figure S7. Full-length gel-electrophoresis images for CTSAI verification experiments. Figure S8.Three-way alignments with Chlamydomonas homologs. Figure S9. HISAT2-Ballgown protocol schematic. Document S1. Exon coordinates for the manually curated HISAT2 candidates. [file 12864_2023_9558_MOESM1_ESM.zip › 12864_2023_9558_MOESM1_ESM.pdf]

**Supplemental Figure Legends:**

Figure S1: Verification gene and transcript expression within replicates
Figure S2: Verification of differences in gene and transcript expression between cell types
Figure S3: Alternate restriction criteria: all genes with two transcripts
Figure S4: Alternate restriction criteria: two oppositely expressed transcripts with large biases
Figure S5: Hand Curation Examples
Figure S6: Convergent Transcription Examples
Figure S7: Full-length gel-electrophoresis images for CTSAI verification experiments
Figure S8: Three-way alignments with *Chlamydomonas* homologs
Figure S9: HISAT2-Ballgown protocol schematic

**Supplemental Document Legends**:

Document S1: Exon coordinates for the manually curated HISAT2 candidates.

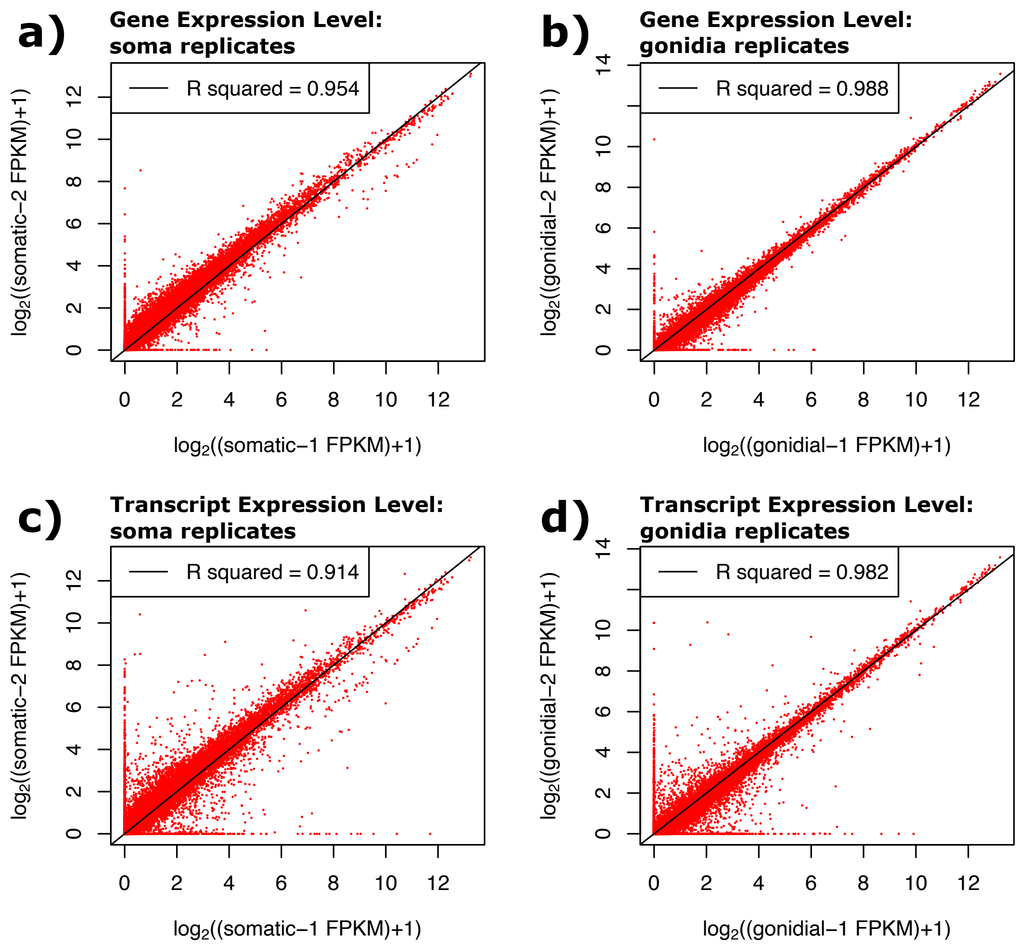


**Supplemental Figure S1**. (**a,b**) Comparison of gene expression levels for somatic and gonidial replicates. Red dots are individual genes. (**c,d**) Comparison of transcript expression levels for somatic and gonidial replicates. Red dots are individual transcripts. We added 1 to the FPKM values because the log is ill-defined when the FPKM is zero.

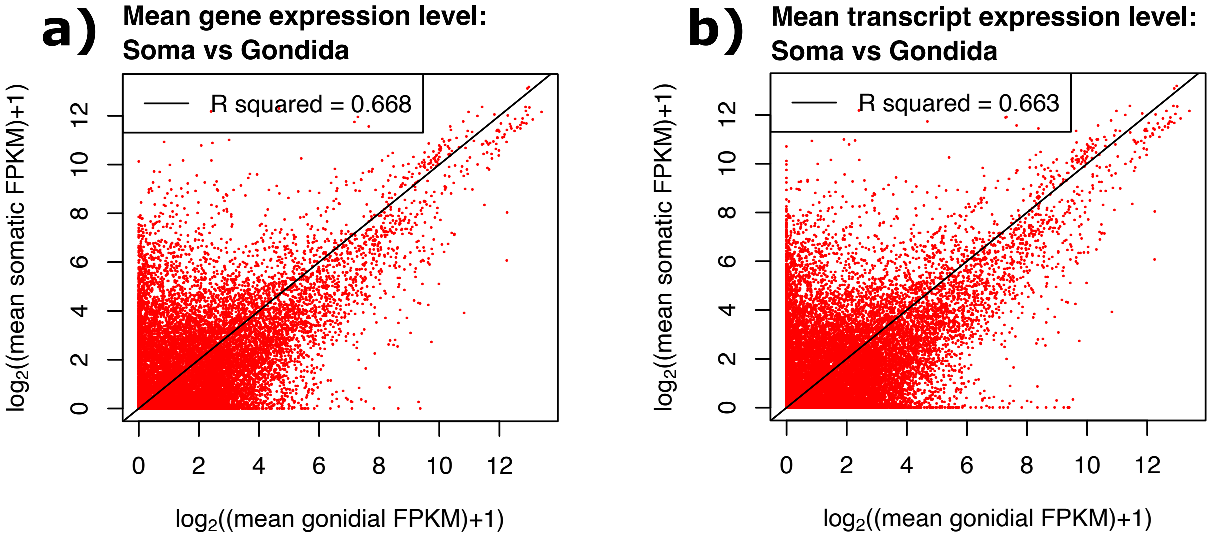


**Supplemental Figure S2:** (**a**) Gene expression levels (log FPKM values) show low correlation between somatic vs. gonidial cells. Red dots are individual genes. (**b**) Transcript expression levels show low correlation between somatic vs. gonidial cells. Red dots are individual transcripts.

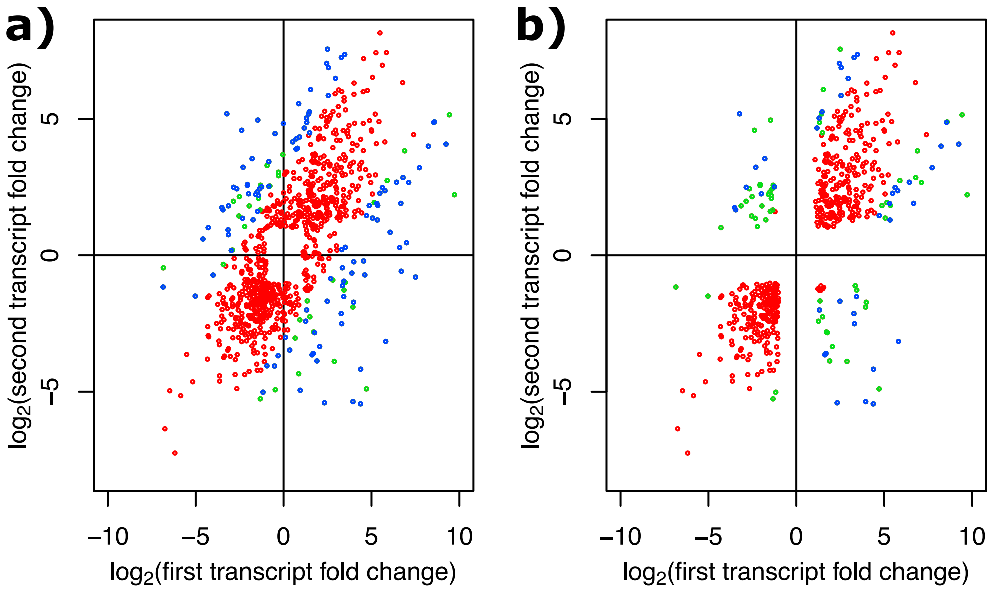


**Supplemental Figure S3:** Log fold change (fc = expression in soma over gonidia) for second vs. first transcript in all genes with two transcripts. Colors indicate the degree of restriction of each gene – blue is the most restricted (passed all three criteria), green follows (passed the first two criteria), and red is the least restricted (only passed the first criterion). Both plots share the last two criteria, which are that the ratio of expression ratios must be greater than 8 and the p-values for each transcript are less than 0.1 respectively. The two plots differ in the first criterion applied. (**a**) The first implemented criterion is that at least one soma-gonidial expression ratio is greater than 2 (or less than 1/2). This set of criteria yields 863 possible genes with the first filter, 145 genes with the second filter, and 110 genes with the final filter. (**b**) The first implemented criterion is that both soma-gonidial expression ratios greater than 2 (and/or less than 1/2). This set of criteria yields 552 possible genes with the first filter, 96 genes with the second filter, and 41 genes with the final filter. Genes far from the diagonal are candidates for CTSAS.

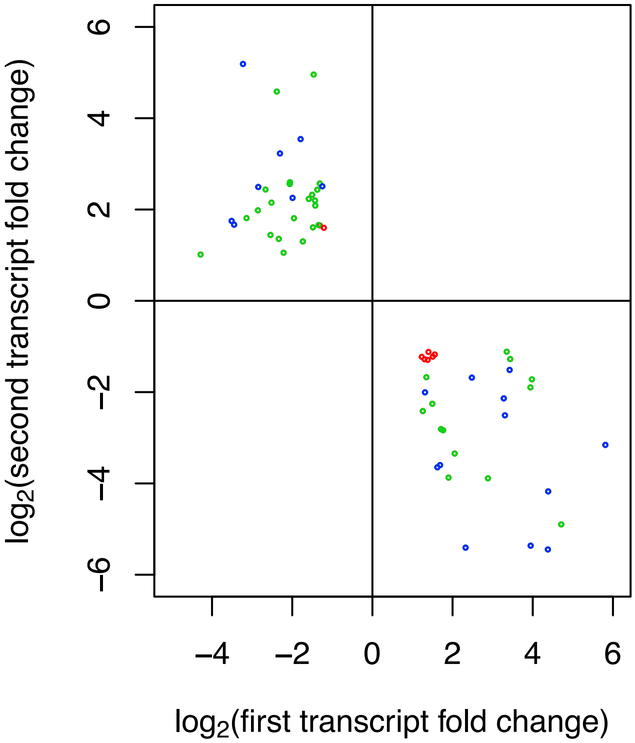


**Supplemental Figure S4**: Log fold change (fc = expression in soma over gonidia) for second vs. first transcript in genes with two transcripts and opposite cell-type bias. Colors indicate the degree of restriction of each gene – blue is the most restricted (passed all three criteria), green follows (passed the first two criteria), and red is the least restricted (only passed the first criterion). The first implemented criterion is that both soma-gonidial expression ratio greater than 2 (or less than 1/2), yielding 63 possible genes. The second implemented criterion is that the ratio of expression ratios is greater than 8, yielding 55 possible genes along with criterion (a). The final implemented criterion is that the p-values for each transcript are less than 0.1, yielding 20 candidate genes along with criteria (a) and (b). Genes far from the diagonal are candidates for CTSAS.

# Manual Curation Examples

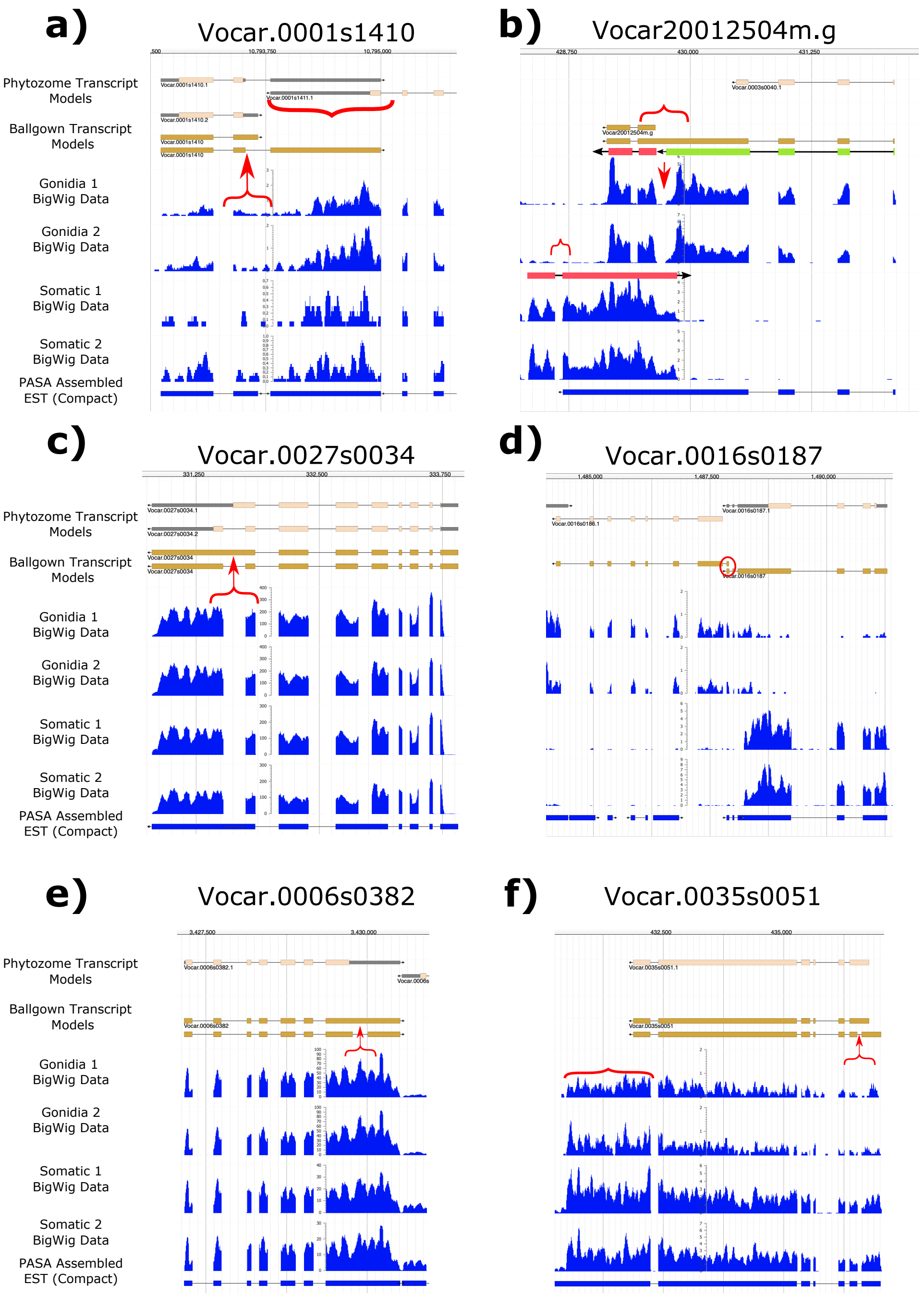


**Supplemental Figure S5:** Example genes that were removed from the analysis during manual curation, with their respective ballgown transcript models, Phytozome transcript models (if present), and read expression data. **(a)** Vocar.0001s1410 was removed due to low coverage in the alternatively-spliced on marked by the red bracket and arrow. The domain marked with the large red bracket is an overlap region between two adjacent genes, so the high expression in the four

samples in this region may not be due to the gene Vocar.0001s1410, but rather its neighbor Vocar.0001s1411. **(b)** Vocar.20012504m.g was removed due to little evidence that the large, central exon of the central transcript was present due to the gap in expression data marked by the red arrow, the extended expression data to the left of the model implying that the model lacked some pieces, and the fact that the expression data, as presented, implied the existence of different transcript models. Looking carefully at the clear splice sites and the areas where the expression tapers, alternative transcript models were proposed: the green model is a separate gene, and the two red models are convergent transcripts over the same locus. **(c)** Vocar.0027s0034 was removed because of the clear lack of evidence of the existence of an alternative splicing region in the 3' end exon, marked by the red bracket. **(d)** Vocar.0016s0187 was removed because ballgown added an additional exon to the top transcript (circled in red) and misidentified two neighboring genes as two transcripts of the same gene because of the created overlap. **(e)** Vocar.0006s0382 was removed because of the clear lack of an alternative splicing site proposed by ballgown in the 3' end exon marked by the red bracket and arrow. **(f)** Vocar.0035s0051 was removed because there the expression data in Gonidia was too weak in order to determine if the alternative splicing site at the 5' end of the transcripts was present or not. These models also seem to be lacking an extension to the 3' end, as evinced by the clear expression data that extends beyond the 3' end.

# Convergent Transcription Examples

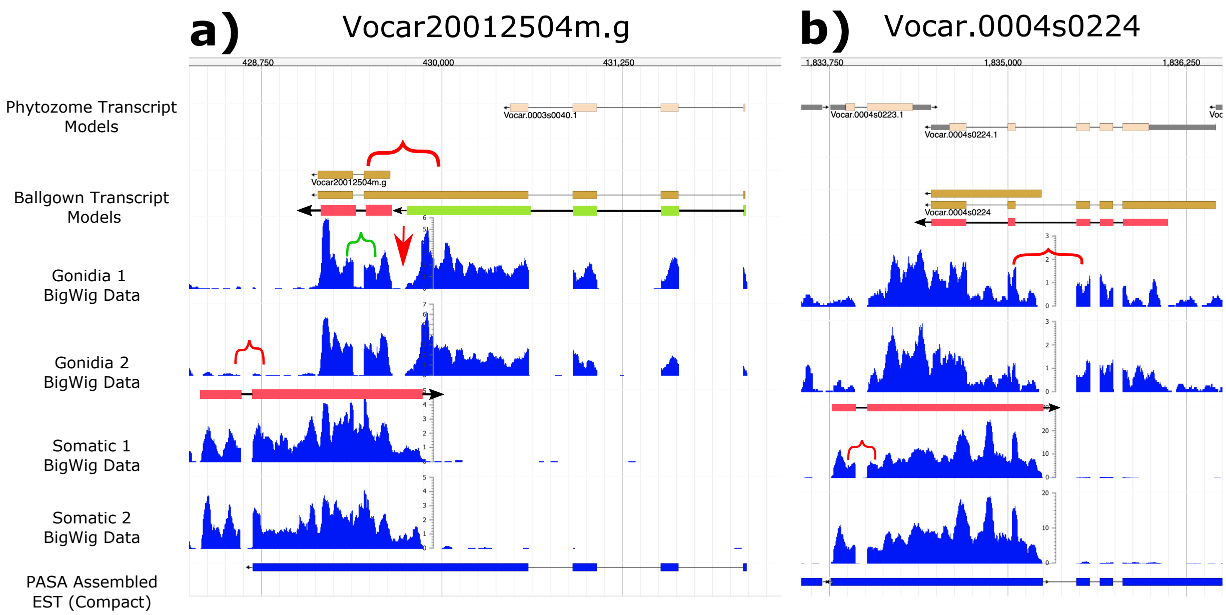


**Supplemental Figure S6:** Examples of convergent transcription in *V. carteri*. In these cases, one gene has a transcript going in the forward-sense direction, while the other transcript goes in the anti-sense direction. Since both Vocar.20012504m.g and Vocar.0004s0224 had misconstructed gene and transcript models, new models were made in red. Since convergent transcription is not CTSAS by our definition, both these cases were removed from the analysis, although they are worth mentioning as an alternative mechanism of producing multiple cell-type specific transcripts. (a) Vocar20012504m.g displayed with its Ballgown models, its expression data, and the hand-made models based on the expression data. In the gonidial samples, a tapering of in gene expression can be seen on either side of the gap identified with the red arrow, which implies that the units of expression to the left and to the right of this gap are in fact separate transcripts, disagreeing with the models Ballgown proposed. Then, checking the steep drops in expression on either side of the gaps in expression in the gonidial samples, we found the GTA/GTG motif (5' splice site) on the right side of each gap, and the CAG/TAG motif (3' splice site) on the left of each gap, implying that the two transcripts in the gonidial samples go in the antisense direction. Meanwhile, the splice site in the somatic expression data (marked with the small red bracket) had the GTA/GTG motif (5' splice site) on the left side of the gap, and the CAG/TAG motif (3' splice site) on the right, implying that that transcript went in the sense direction. Overall, the two transcripts that overlapped were taken to be the alternative, convergent transcripts of the Vocar.20012504m.g gene (marked in red), while the third transcript (in green) was thought to be a separate gene. (b) Vocar.0004s0224 is another case of misidentified convergent transcription. Using the same method of identifying the motifs flanking each

potential splice site, we determined that the gonidial transcript goes in the antisense direction, while the somatic transcript goes in the sense direction (in red). It is possible that this is a case where Phytozome misidentified two convergent transcripts as two different genes, as evinced by the two Phytozome gene models at the top of the panel which overlap the expression data.

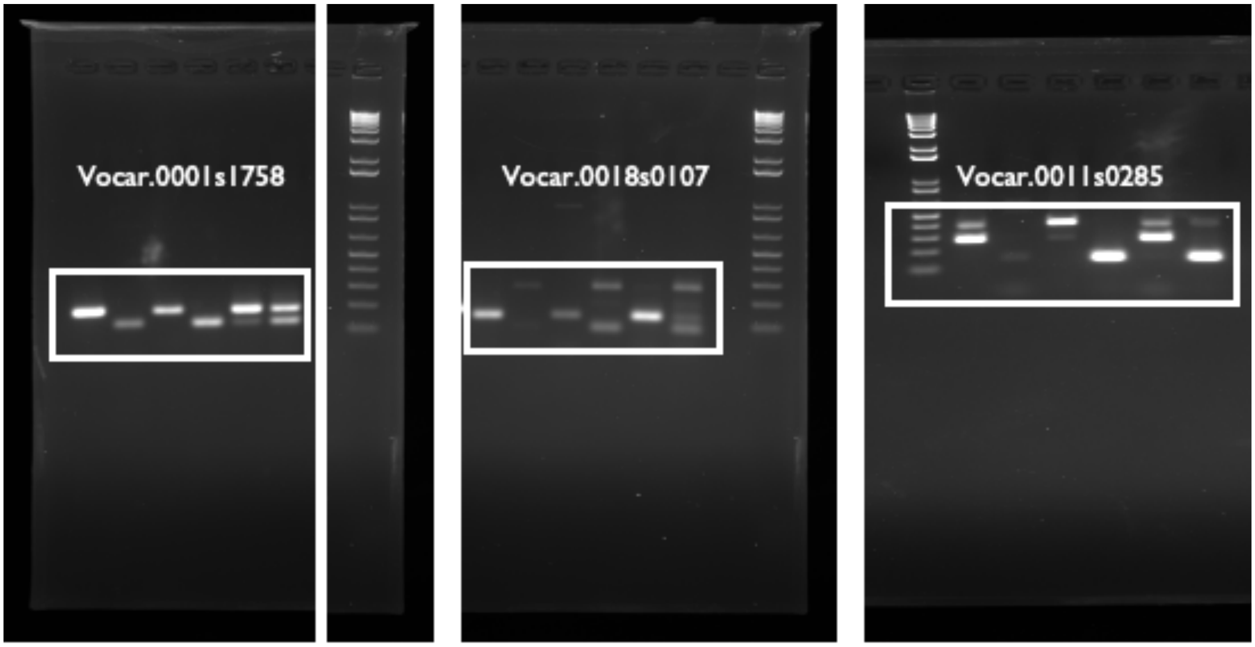


**Supplemental Figure S7**: Full gels for CTSAI verification experiments. All white boxes are the areas cropped for Figure 4 in the main text. Experiments for Vocar.0001s1758, Vocar.0011s0285, and Vocar.0018s0107 are successful. Vocar.0001s1758 and Vocar.0018s0107 were run on the same gel, while Vocar.0011s0285 was run on a separate gel. Non-relevant middle lanes were cropped out between the markers for Vocar.0001s1758 and Vocar.0018s0107. The shared ladder for these two genes is aligned next to both these genes.

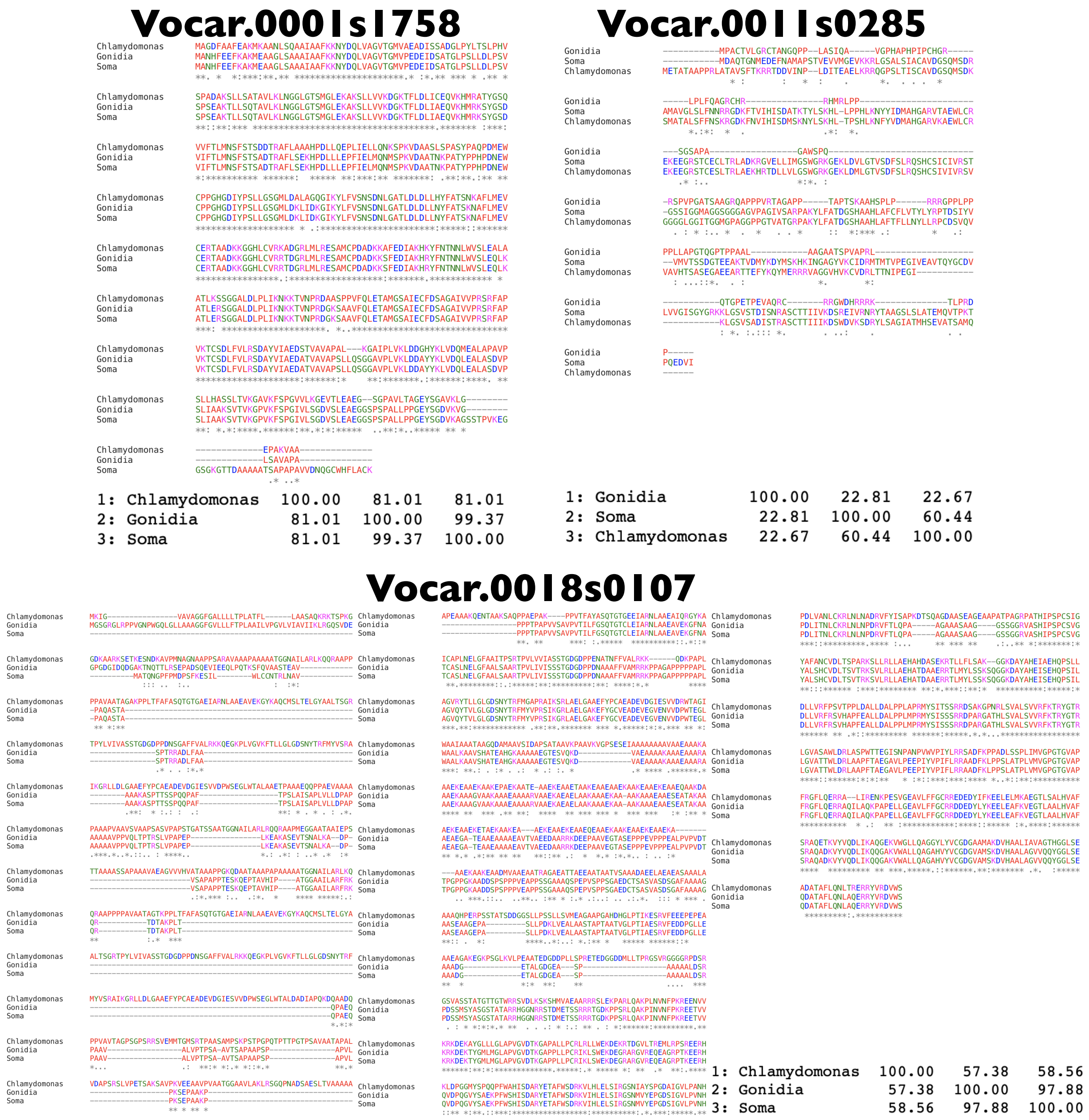


**Supplemental Figure S8:** Three-Way alignment of the two isoforms (listed by the cell-type in which they are most expressed) and their Chlamydomonas ortholog. Vocar.0001s1758's isoforms are very similar to the original Chlamydomonas sequence and have similar lengths. The 3' end of the somatic isoform is different in length from that of the gonidial isoform and Chlamydomonas, while the gonidial and Chlamydomonas isoform are similar in length but differ by a few amino acids. Thus the gonidial isoform may be the more ancestral transcript. Vocar.0011s0285's isoforms are very different from the Chlamydomonas homolog (only ~22-23% similarity). The exon structure of the gonidial isoform is very different from that of the other two isoforms. It is probable that the Volvox sequence diverged from Chlamydomonas and

then became two separate isoforms. Vocar.0018s0107's isoforms differ at the 5' end, but have mostly similar exon structure from that point forward. Meanwhile, there appear to be large regions where the Volvox isoforms sequences do not line up with the Chlamydomonas sequence. It is unclear which isoform is more ancestral, however the somatic isoform has a higher percent similarity than the gonidial isoform.

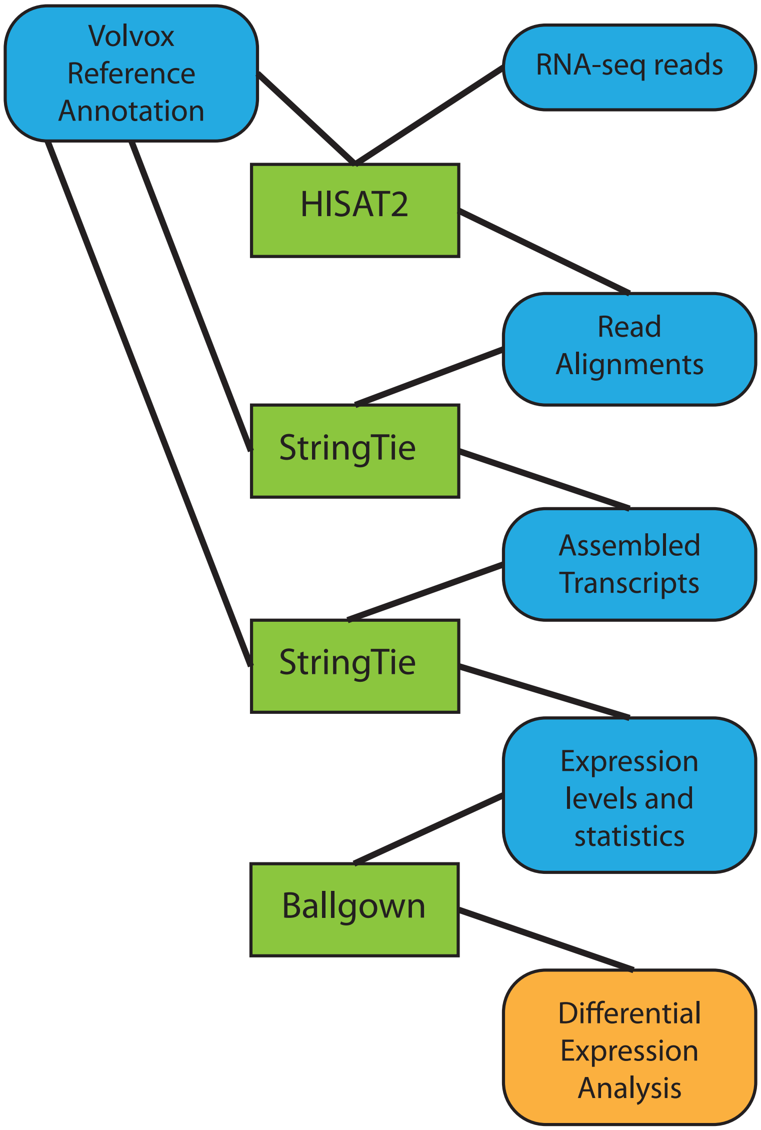


**Supplemental Figure S9:** Schematic of the bioinformatic analysis using the HISAT2, String Tie, and Ballgown software packages [Pertea et al., 2016]. The workflow is from top to bottom.
