# Supplementary material for: Identification of cell-type specific alternative transcripts in the multicellular alga Volvox carteri
Source: BMC Genomics. 2023 Oct 30;24:654. doi: 10.1186/s12864-023-09558-0 (PMC10617192; doi:10.1186/s12864-023-09558-0)
Supplement: Supplementary file 1 — Additional file 1: Figure S1. Verification gene and transcript expression within replicates. Figure S2. Verification of differences in gene and transcript expression between cell types. Figure S3. Alternate restriction criteria: all genes with two transcripts. Figure S4. Alternate restriction criteria: two oppositely expressed transcripts with large biases. Figure S5. Hand Curation Examples. Figure S6. Convergent Transcription Examples. Figure S7. Full-length gel-electrophoresis images for CTSAI verification experiments. Figure S8.Three-way alignments with Chlamydomonas homologs. Figure S9. HISAT2-Ballgown protocol schematic. Document S1. Exon coordinates for the manually curated HISAT2 candidates. [file 12864_2023_9558_MOESM1_ESM.zip › SupplementalDocS1_Exons_HISAT2_Candidates_ESM.docx]

scaffold_1 StringTie transcript 2341677 2344887 1000 - . gene_id "MSTRG.245"; transcript_id "PAC4GC:715031"; gene_name "Vocar.0001s0311"; ref_gene_id "Vocar.0001s0311";

scaffold_1 StringTie exon 2341677 2343186 1000 - . gene_id "MSTRG.245"; transcript_id "PAC4GC:715031"; exon_number "1"; gene_name "Vocar.0001s0311"; ref_gene_id "Vocar.0001s0311";

scaffold_1 StringTie exon 2343886 2344033 1000 - . gene_id "MSTRG.245"; transcript_id "PAC4GC:715031"; exon_number "2"; gene_name "Vocar.0001s0311"; ref_gene_id "Vocar.0001s0311";

scaffold_1 StringTie exon 2344485 2344541 1000 - . gene_id "MSTRG.245"; transcript_id "PAC4GC:715031"; exon_number "3"; gene_name "Vocar.0001s0311"; ref_gene_id "Vocar.0001s0311";

scaffold_1 StringTie exon 2344641 2344887 1000 - . gene_id "MSTRG.245"; transcript_id "PAC4GC:715031"; exon_number "4"; gene_name "Vocar.0001s0311"; ref_gene_id "Vocar.0001s0311";

scaffold_1 StringTie transcript 2341682 2344887 1000 - . gene_id "MSTRG.245"; transcript_id "MSTRG.245.2";

scaffold_1 StringTie exon 2341682 2343013 1000 - . gene_id "MSTRG.245"; transcript_id "MSTRG.245.2"; exon_number "1";

scaffold_1 StringTie exon 2343059 2343186 1000 - . gene_id "MSTRG.245"; transcript_id "MSTRG.245.2"; exon_number "2";

scaffold_1 StringTie exon 2343886 2344033 1000 - . gene_id "MSTRG.245"; transcript_id "MSTRG.245.2"; exon_number "3";

scaffold_1 StringTie exon 2344485 2344541 1000 - . gene_id "MSTRG.245"; transcript_id "MSTRG.245.2"; exon_number "4";

scaffold_1 StringTie exon 2344641 2344887 1000 - . gene_id "MSTRG.245"; transcript_id "MSTRG.245.2"; exon_number "5";

scaffold_1 StringTie transcript 13542209 13547795 1000 + . gene_id "MSTRG.1368"; transcript_id "PAC4GC:714674"; gene_name "Vocar.0001s1758"; ref_gene_id "Vocar.0001s1758";

scaffold_1 StringTie exon 13542209 13542385 1000 + . gene_id "MSTRG.1368"; transcript_id "PAC4GC:714674"; exon_number "1"; gene_name "Vocar.0001s1758"; ref_gene_id "Vocar.0001s1758";

scaffold_1 StringTie exon 13542457 13542567 1000 + . gene_id "MSTRG.1368"; transcript_id "PAC4GC:714674"; exon_number "2"; gene_name "Vocar.0001s1758"; ref_gene_id "Vocar.0001s1758";

scaffold_1 StringTie exon 13542790 13542870 1000 + . gene_id "MSTRG.1368"; transcript_id "PAC4GC:714674"; exon_number "3"; gene_name "Vocar.0001s1758"; ref_gene_id "Vocar.0001s1758";

scaffold_1 StringTie exon 13543384 13543512 1000 + . gene_id "MSTRG.1368"; transcript_id "PAC4GC:714674"; exon_number "4"; gene_name "Vocar.0001s1758"; ref_gene_id "Vocar.0001s1758";

scaffold_1 StringTie exon 13543634 13543750 1000 + . gene_id "MSTRG.1368"; transcript_id "PAC4GC:714674"; exon_number "5"; gene_name "Vocar.0001s1758"; ref_gene_id "Vocar.0001s1758";

scaffold_1 StringTie exon 13544345 13544439 1000 + . gene_id "MSTRG.1368"; transcript_id "PAC4GC:714674"; exon_number "6"; gene_name "Vocar.0001s1758"; ref_gene_id "Vocar.0001s1758";

scaffold_1 StringTie exon 13544847 13544927 1000 + . gene_id "MSTRG.1368"; transcript_id "PAC4GC:714674"; exon_number "7"; gene_name "Vocar.0001s1758"; ref_gene_id "Vocar.0001s1758";

scaffold_1 StringTie exon 13545348 13545444 1000 + . gene_id "MSTRG.1368"; transcript_id "PAC4GC:714674"; exon_number "8"; gene_name "Vocar.0001s1758"; ref_gene_id "Vocar.0001s1758";

scaffold_1 StringTie exon 13545551 13545687 1000 + . gene_id "MSTRG.1368"; transcript_id "PAC4GC:714674"; exon_number "9"; gene_name "Vocar.0001s1758"; ref_gene_id "Vocar.0001s1758";

scaffold_1 StringTie exon 13545999 13546189 1000 + . gene_id "MSTRG.1368"; transcript_id "PAC4GC:714674"; exon_number "10"; gene_name "Vocar.0001s1758"; ref_gene_id "Vocar.0001s1758";

scaffold_1 StringTie exon 13546447 13546640 1000 + . gene_id "MSTRG.1368"; transcript_id "PAC4GC:714674"; exon_number "11"; gene_name "Vocar.0001s1758"; ref_gene_id "Vocar.0001s1758";

scaffold_1 StringTie exon 13546875 13546950 1000 + . gene_id "MSTRG.1368"; transcript_id "PAC4GC:714674"; exon_number "12"; gene_name "Vocar.0001s1758"; ref_gene_id "Vocar.0001s1758";

scaffold_1 StringTie exon 13547169 13547795 1000 + . gene_id "MSTRG.1368"; transcript_id "PAC4GC:714674"; exon_number "13"; gene_name "Vocar.0001s1758"; ref_gene_id "Vocar.0001s1758";

scaffold_1 StringTie transcript 13542209 13550813 1000 + . gene_id "MSTRG.1368"; transcript_id "PAC4GC:714673"; gene_name "Vocar.0001s1758"; ref_gene_id "Vocar.0001s1758";

scaffold_1 StringTie exon 13542209 13542385 1000 + . gene_id "MSTRG.1368"; transcript_id "PAC4GC:714673"; exon_number "1"; gene_name "Vocar.0001s1758"; ref_gene_id "Vocar.0001s1758";

scaffold_1 StringTie exon 13542457 13542567 1000 + . gene_id "MSTRG.1368"; transcript_id "PAC4GC:714673"; exon_number "2"; gene_name "Vocar.0001s1758"; ref_gene_id "Vocar.0001s1758";

scaffold_1 StringTie exon 13542790 13542870 1000 + . gene_id "MSTRG.1368"; transcript_id "PAC4GC:714673"; exon_number "3"; gene_name "Vocar.0001s1758"; ref_gene_id "Vocar.0001s1758";

scaffold_1 StringTie exon 13543384 13543512 1000 + . gene_id "MSTRG.1368"; transcript_id "PAC4GC:714673"; exon_number "4"; gene_name "Vocar.0001s1758"; ref_gene_id "Vocar.0001s1758";

scaffold_1 StringTie exon 13543634 13543750 1000 + . gene_id "MSTRG.1368"; transcript_id "PAC4GC:714673"; exon_number "5"; gene_name "Vocar.0001s1758"; ref_gene_id "Vocar.0001s1758";

scaffold_1 StringTie exon 13544345 13544439 1000 + . gene_id "MSTRG.1368"; transcript_id "PAC4GC:714673"; exon_number "6"; gene_name "Vocar.0001s1758"; ref_gene_id "Vocar.0001s1758";

scaffold_1 StringTie exon 13544847 13544927 1000 + . gene_id "MSTRG.1368"; transcript_id "PAC4GC:714673"; exon_number "7"; gene_name "Vocar.0001s1758"; ref_gene_id "Vocar.0001s1758";

scaffold_1 StringTie exon 13545348 13545444 1000 + . gene_id "MSTRG.1368"; transcript_id "PAC4GC:714673"; exon_number "8"; gene_name "Vocar.0001s1758"; ref_gene_id "Vocar.0001s1758";

scaffold_1 StringTie exon 13545551 13545687 1000 + . gene_id "MSTRG.1368"; transcript_id "PAC4GC:714673"; exon_number "9"; gene_name "Vocar.0001s1758"; ref_gene_id "Vocar.0001s1758";

scaffold_1 StringTie exon 13545999 13546189 1000 + . gene_id "MSTRG.1368"; transcript_id "PAC4GC:714673"; exon_number "10"; gene_name "Vocar.0001s1758"; ref_gene_id "Vocar.0001s1758";

scaffold_1 StringTie exon 13546447 13546640 1000 + . gene_id "MSTRG.1368"; transcript_id "PAC4GC:714673"; exon_number "11"; gene_name "Vocar.0001s1758"; ref_gene_id "Vocar.0001s1758";

scaffold_1 StringTie exon 13546875 13546950 1000 + . gene_id "MSTRG.1368"; transcript_id "PAC4GC:714673"; exon_number "12"; gene_name "Vocar.0001s1758"; ref_gene_id "Vocar.0001s1758";

scaffold_1 StringTie exon 13547169 13547263 1000 + . gene_id "MSTRG.1368"; transcript_id "PAC4GC:714673"; exon_number "13"; gene_name "Vocar.0001s1758"; ref_gene_id "Vocar.0001s1758";

scaffold_1 StringTie exon 13549326 13550813 1000 + . gene_id "MSTRG.1368"; transcript_id "PAC4GC:714673"; exon_number "14"; gene_name "Vocar.0001s1758"; ref_gene_id "Vocar.0001s1758";

scaffold_11 StringTie transcript 2321358 2327887 1000 + . gene_id "MSTRG.1661"; transcript_id "PAC4GC:724672"; gene_name "Vocar.0011s0285"; ref_gene_id "Vocar.0011s0285";

scaffold_11 StringTie exon 2321358 2321729 1000 + . gene_id "MSTRG.1661"; transcript_id "PAC4GC:724672"; exon_number "1"; gene_name "Vocar.0011s0285"; ref_gene_id "Vocar.0011s0285";

scaffold_11 StringTie exon 2322002 2322116 1000 + . gene_id "MSTRG.1661"; transcript_id "PAC4GC:724672"; exon_number "2"; gene_name "Vocar.0011s0285"; ref_gene_id "Vocar.0011s0285";

scaffold_11 StringTie exon 2322263 2322393 1000 + . gene_id "MSTRG.1661"; transcript_id "PAC4GC:724672"; exon_number "3"; gene_name "Vocar.0011s0285"; ref_gene_id "Vocar.0011s0285";

scaffold_11 StringTie exon 2322950 2323168 1000 + . gene_id "MSTRG.1661"; transcript_id "PAC4GC:724672"; exon_number "4"; gene_name "Vocar.0011s0285"; ref_gene_id "Vocar.0011s0285";

scaffold_11 StringTie exon 2323661 2323769 1000 + . gene_id "MSTRG.1661"; transcript_id "PAC4GC:724672"; exon_number "5"; gene_name "Vocar.0011s0285"; ref_gene_id "Vocar.0011s0285";

scaffold_11 StringTie exon 2324206 2324327 1000 + . gene_id "MSTRG.1661"; transcript_id "PAC4GC:724672"; exon_number "6"; gene_name "Vocar.0011s0285"; ref_gene_id "Vocar.0011s0285";

scaffold_11 StringTie exon 2324804 2324873 1000 + . gene_id "MSTRG.1661"; transcript_id "PAC4GC:724672"; exon_number "7"; gene_name "Vocar.0011s0285"; ref_gene_id "Vocar.0011s0285";

scaffold_11 StringTie exon 2325285 2327887 1000 + . gene_id "MSTRG.1661"; transcript_id "PAC4GC:724672"; exon_number "8"; gene_name "Vocar.0011s0285"; ref_gene_id "Vocar.0011s0285";

scaffold_11 StringTie transcript 2324356 2327884 1000 + . gene_id "MSTRG.1661"; transcript_id "MSTRG.1661.2";

scaffold_11 StringTie exon 2324356 2324677 1000 + . gene_id "MSTRG.1661"; transcript_id "MSTRG.1661.2"; exon_number "1";

scaffold_11 StringTie exon 2324804 2324873 1000 + . gene_id "MSTRG.1661"; transcript_id "MSTRG.1661.2"; exon_number "2";

scaffold_11 StringTie exon 2325285 2327884 1000 + . gene_id "MSTRG.1661"; transcript_id "MSTRG.1661.2"; exon_number "3";

scaffold_18 StringTie transcript 813935 823398 1000 + . gene_id "MSTRG.3211"; transcript_id "PAC4GC:728217"; gene_name "Vocar.0018s0107"; ref_gene_id "Vocar.0018s0107";

scaffold_18 StringTie exon 813935 814387 1000 + . gene_id "MSTRG.3211"; transcript_id "PAC4GC:728217"; exon_number "1"; gene_name "Vocar.0018s0107"; ref_gene_id "Vocar.0018s0107";

scaffold_18 StringTie exon 815191 816255 1000 + . gene_id "MSTRG.3211"; transcript_id "PAC4GC:728217"; exon_number "2"; gene_name "Vocar.0018s0107"; ref_gene_id "Vocar.0018s0107";

scaffold_18 StringTie exon 816605 817560 1000 + . gene_id "MSTRG.3211"; transcript_id "PAC4GC:728217"; exon_number "3"; gene_name "Vocar.0018s0107"; ref_gene_id "Vocar.0018s0107";

scaffold_18 StringTie exon 818044 818143 1000 + . gene_id "MSTRG.3211"; transcript_id "PAC4GC:728217"; exon_number "4"; gene_name "Vocar.0018s0107"; ref_gene_id "Vocar.0018s0107";

scaffold_18 StringTie exon 818593 818828 1000 + . gene_id "MSTRG.3211"; transcript_id "PAC4GC:728217"; exon_number "5"; gene_name "Vocar.0018s0107"; ref_gene_id "Vocar.0018s0107";

scaffold_18 StringTie exon 819311 819698 1000 + . gene_id "MSTRG.3211"; transcript_id "PAC4GC:728217"; exon_number "6"; gene_name "Vocar.0018s0107"; ref_gene_id "Vocar.0018s0107";

scaffold_18 StringTie exon 820107 820333 1000 + . gene_id "MSTRG.3211"; transcript_id "PAC4GC:728217"; exon_number "7"; gene_name "Vocar.0018s0107"; ref_gene_id "Vocar.0018s0107";

scaffold_18 StringTie exon 820541 820781 1000 + . gene_id "MSTRG.3211"; transcript_id "PAC4GC:728217"; exon_number "8"; gene_name "Vocar.0018s0107"; ref_gene_id "Vocar.0018s0107";

scaffold_18 StringTie exon 821177 821341 1000 + . gene_id "MSTRG.3211"; transcript_id "PAC4GC:728217"; exon_number "9"; gene_name "Vocar.0018s0107"; ref_gene_id "Vocar.0018s0107";

scaffold_18 StringTie exon 821768 821938 1000 + . gene_id "MSTRG.3211"; transcript_id "PAC4GC:728217"; exon_number "10"; gene_name "Vocar.0018s0107"; ref_gene_id "Vocar.0018s0107";

scaffold_18 StringTie exon 822253 823398 1000 + . gene_id "MSTRG.3211"; transcript_id "PAC4GC:728217"; exon_number "11"; gene_name "Vocar.0018s0107"; ref_gene_id "Vocar.0018s0107";

scaffold_18 StringTie transcript 814746 823398 1000 + . gene_id "MSTRG.3211"; transcript_id "PAC4GC:728218"; gene_name "Vocar.0018s0107"; ref_gene_id "Vocar.0018s0107";

scaffold_18 StringTie exon 814746 815000 1000 + . gene_id "MSTRG.3211"; transcript_id "PAC4GC:728218"; exon_number "1"; gene_name "Vocar.0018s0107"; ref_gene_id "Vocar.0018s0107";

scaffold_18 StringTie exon 815191 816255 1000 + . gene_id "MSTRG.3211"; transcript_id "PAC4GC:728218"; exon_number "2"; gene_name "Vocar.0018s0107"; ref_gene_id "Vocar.0018s0107";

scaffold_18 StringTie exon 816605 817560 1000 + . gene_id "MSTRG.3211"; transcript_id "PAC4GC:728218"; exon_number "3"; gene_name "Vocar.0018s0107"; ref_gene_id "Vocar.0018s0107";

scaffold_18 StringTie exon 818044 818143 1000 + . gene_id "MSTRG.3211"; transcript_id "PAC4GC:728218"; exon_number "4"; gene_name "Vocar.0018s0107"; ref_gene_id "Vocar.0018s0107";

scaffold_18 StringTie exon 818593 818828 1000 + . gene_id "MSTRG.3211"; transcript_id "PAC4GC:728218"; exon_number "5"; gene_name "Vocar.0018s0107"; ref_gene_id "Vocar.0018s0107";

scaffold_18 StringTie exon 819311 819698 1000 + . gene_id "MSTRG.3211"; transcript_id "PAC4GC:728218"; exon_number "6"; gene_name "Vocar.0018s0107"; ref_gene_id "Vocar.0018s0107";

scaffold_18 StringTie exon 820107 820333 1000 + . gene_id "MSTRG.3211"; transcript_id "PAC4GC:728218"; exon_number "7"; gene_name "Vocar.0018s0107"; ref_gene_id "Vocar.0018s0107";

scaffold_18 StringTie exon 820541 820781 1000 + . gene_id "MSTRG.3211"; transcript_id "PAC4GC:728218"; exon_number "8"; gene_name "Vocar.0018s0107"; ref_gene_id "Vocar.0018s0107";

scaffold_18 StringTie exon 821177 821341 1000 + . gene_id "MSTRG.3211"; transcript_id "PAC4GC:728218"; exon_number "9"; gene_name "Vocar.0018s0107"; ref_gene_id "Vocar.0018s0107";

scaffold_18 StringTie exon 821768 821938 1000 + . gene_id "MSTRG.3211"; transcript_id "PAC4GC:728218"; exon_number "10"; gene_name "Vocar.0018s0107"; ref_gene_id "Vocar.0018s0107";

scaffold_18 StringTie exon 822253 823398 1000 + . gene_id "MSTRG.3211"; transcript_id "PAC4GC:728218"; exon_number "11"; gene_name "Vocar.0018s0107"; ref_gene_id "Vocar.0018s0107";

scaffold_25 StringTie transcript 1863730 1864125 1000 - . gene_id "MSTRG.5198"; transcript_id "MSTRG.5198.1";

scaffold_25 StringTie exon 1863730 1863773 1000 - . gene_id "MSTRG.5198"; transcript_id "MSTRG.5198.1"; exon_number "1";

scaffold_25 StringTie exon 1863944 1864125 1000 - . gene_id "MSTRG.5198"; transcript_id "MSTRG.5198.1"; exon_number "2";

scaffold_25 StringTie transcript 1863740 1868521 1000 - . gene_id "MSTRG.5198"; transcript_id "MSTRG.5198.2";

scaffold_25 StringTie exon 1863740 1863773 1000 - . gene_id "MSTRG.5198"; transcript_id "MSTRG.5198.2"; exon_number "1";

scaffold_25 StringTie exon 1864842 1864967 1000 - . gene_id "MSTRG.5198"; transcript_id "MSTRG.5198.2"; exon_number "2";

scaffold_25 StringTie exon 1865381 1865669 1000 - . gene_id "MSTRG.5198"; transcript_id "MSTRG.5198.2"; exon_number "3";

scaffold_25 StringTie exon 1866225 1866350 1000 - . gene_id "MSTRG.5198"; transcript_id "MSTRG.5198.2"; exon_number "4";

scaffold_25 StringTie exon 1866706 1866865 1000 - . gene_id "MSTRG.5198"; transcript_id "MSTRG.5198.2"; exon_number "5";

scaffold_25 StringTie exon 1867293 1867411 1000 - . gene_id "MSTRG.5198"; transcript_id "MSTRG.5198.2"; exon_number "6";

scaffold_25 StringTie exon 1868078 1868156 1000 - . gene_id "MSTRG.5198"; transcript_id "MSTRG.5198.2"; exon_number "7";

scaffold_25 StringTie exon 1868223 1868521 1000 - . gene_id "MSTRG.5198"; transcript_id "MSTRG.5198.2"; exon_number "8";

scaffold_39 StringTie transcript 832092 836377 1000 - . gene_id "MSTRG.7312"; transcript_id "PAC4GC:727415"; gene_name "Vocar.0039s0085"; ref_gene_id "Vocar.0039s0085";

scaffold_39 StringTie exon 832092 833472 1000 - . gene_id "MSTRG.7312"; transcript_id "PAC4GC:727415"; exon_number "1"; gene_name "Vocar.0039s0085"; ref_gene_id "Vocar.0039s0085";

scaffold_39 StringTie exon 833675 833920 1000 - . gene_id "MSTRG.7312"; transcript_id "PAC4GC:727415"; exon_number "2"; gene_name "Vocar.0039s0085"; ref_gene_id "Vocar.0039s0085";

scaffold_39 StringTie exon 834104 834233 1000 - . gene_id "MSTRG.7312"; transcript_id "PAC4GC:727415"; exon_number "3"; gene_name "Vocar.0039s0085"; ref_gene_id "Vocar.0039s0085";

scaffold_39 StringTie exon 834442 834613 1000 - . gene_id "MSTRG.7312"; transcript_id "PAC4GC:727415"; exon_number "4"; gene_name "Vocar.0039s0085"; ref_gene_id "Vocar.0039s0085";

scaffold_39 StringTie exon 834774 835754 1000 - . gene_id "MSTRG.7312"; transcript_id "PAC4GC:727415"; exon_number "5"; gene_name "Vocar.0039s0085"; ref_gene_id "Vocar.0039s0085";

scaffold_39 StringTie exon 836071 836377 1000 - . gene_id "MSTRG.7312"; transcript_id "PAC4GC:727415"; exon_number "6"; gene_name "Vocar.0039s0085"; ref_gene_id "Vocar.0039s0085";

scaffold_39 StringTie transcript 832129 834593 1000 - . gene_id "MSTRG.7312"; transcript_id "MSTRG.7312.2";

scaffold_39 StringTie exon 832129 833472 1000 - . gene_id "MSTRG.7312"; transcript_id "MSTRG.7312.2"; exon_number "1";

scaffold_39 StringTie exon 833675 833920 1000 - . gene_id "MSTRG.7312"; transcript_id "MSTRG.7312.2"; exon_number "2";

scaffold_39 StringTie exon 834032 834233 1000 - . gene_id "MSTRG.7312"; transcript_id "MSTRG.7312.2"; exon_number "3";

scaffold_39 StringTie exon 834442 834593 1000 - . gene_id "MSTRG.7312"; transcript_id "MSTRG.7312.2"; exon_number "4";

scaffold_4 StringTie transcript 2368850 2380393 1000 - . gene_id "MSTRG.7556"; transcript_id "PAC4GC:719267"; gene_name "Vocar.0004s0303"; ref_gene_id "Vocar.0004s0303";

scaffold_4 StringTie exon 2368850 2371386 1000 - . gene_id "MSTRG.7556"; transcript_id "PAC4GC:719267"; exon_number "1"; gene_name "Vocar.0004s0303"; ref_gene_id "Vocar.0004s0303";

scaffold_4 StringTie exon 2371492 2371608 1000 - . gene_id "MSTRG.7556"; transcript_id "PAC4GC:719267"; exon_number "2"; gene_name "Vocar.0004s0303"; ref_gene_id "Vocar.0004s0303";

scaffold_4 StringTie exon 2371972 2372003 1000 - . gene_id "MSTRG.7556"; transcript_id "PAC4GC:719267"; exon_number "3"; gene_name "Vocar.0004s0303"; ref_gene_id "Vocar.0004s0303";

scaffold_4 StringTie exon 2372910 2373538 1000 - . gene_id "MSTRG.7556"; transcript_id "PAC4GC:719267"; exon_number "4"; gene_name "Vocar.0004s0303"; ref_gene_id "Vocar.0004s0303";

scaffold_4 StringTie exon 2379910 2379951 1000 - . gene_id "MSTRG.7556"; transcript_id "PAC4GC:719267"; exon_number "5"; gene_name "Vocar.0004s0303"; ref_gene_id "Vocar.0004s0303";

scaffold_4 StringTie exon 2380097 2380393 1000 - . gene_id "MSTRG.7556"; transcript_id "PAC4GC:719267"; exon_number "6"; gene_name "Vocar.0004s0303"; ref_gene_id "Vocar.0004s0303";

scaffold_4 StringTie transcript 2369079 2374108 1000 - . gene_id "MSTRG.7556"; transcript_id "MSTRG.7556.2";

scaffold_4 StringTie exon 2369079 2371386 1000 - . gene_id "MSTRG.7556"; transcript_id "MSTRG.7556.2"; exon_number "1";

scaffold_4 StringTie exon 2371492 2371608 1000 - . gene_id "MSTRG.7556"; transcript_id "MSTRG.7556.2"; exon_number "2";

scaffold_4 StringTie exon 2371972 2372003 1000 - . gene_id "MSTRG.7556"; transcript_id "MSTRG.7556.2"; exon_number "3";

scaffold_4 StringTie exon 2372910 2373538 1000 - . gene_id "MSTRG.7556"; transcript_id "MSTRG.7556.2"; exon_number "4";

scaffold_4 StringTie exon 2373849 2374108 1000 - . gene_id "MSTRG.7556"; transcript_id "MSTRG.7556.2"; exon_number "5";

scaffold_5 StringTie transcript 1875206 1891288 1000 - . gene_id "MSTRG.8537"; transcript_id "MSTRG.8537.1";

scaffold_5 StringTie exon 1875206 1877571 1000 - . gene_id "MSTRG.8537"; transcript_id "MSTRG.8537.1"; exon_number "1";

scaffold_5 StringTie exon 1878123 1878219 1000 - . gene_id "MSTRG.8537"; transcript_id "MSTRG.8537.1"; exon_number "2";

scaffold_5 StringTie exon 1878728 1879886 1000 - . gene_id "MSTRG.8537"; transcript_id "MSTRG.8537.1"; exon_number "3";

scaffold_5 StringTie exon 1879982 1880144 1000 - . gene_id "MSTRG.8537"; transcript_id "MSTRG.8537.1"; exon_number "4";

scaffold_5 StringTie exon 1881474 1881648 1000 - . gene_id "MSTRG.8537"; transcript_id "MSTRG.8537.1"; exon_number "5";

scaffold_5 StringTie exon 1882013 1882173 1000 - . gene_id "MSTRG.8537"; transcript_id "MSTRG.8537.1"; exon_number "6";

scaffold_5 StringTie exon 1883185 1883385 1000 - . gene_id "MSTRG.8537"; transcript_id "MSTRG.8537.1"; exon_number "7";

scaffold_5 StringTie exon 1883453 1885641 1000 - . gene_id "MSTRG.8537"; transcript_id "MSTRG.8537.1"; exon_number "8";

scaffold_5 StringTie exon 1885710 1885779 1000 - . gene_id "MSTRG.8537"; transcript_id "MSTRG.8537.1"; exon_number "9";

scaffold_5 StringTie exon 1885867 1886015 1000 - . gene_id "MSTRG.8537"; transcript_id "MSTRG.8537.1"; exon_number "10";

scaffold_5 StringTie exon 1886089 1888067 1000 - . gene_id "MSTRG.8537"; transcript_id "MSTRG.8537.1"; exon_number "11";

scaffold_5 StringTie exon 1888139 1888184 1000 - . gene_id "MSTRG.8537"; transcript_id "MSTRG.8537.1"; exon_number "12";

scaffold_5 StringTie exon 1888263 1888359 1000 - . gene_id "MSTRG.8537"; transcript_id "MSTRG.8537.1"; exon_number "13";

scaffold_5 StringTie exon 1888430 1888697 1000 - . gene_id "MSTRG.8537"; transcript_id "MSTRG.8537.1"; exon_number "14";

scaffold_5 StringTie exon 1891122 1891288 1000 - . gene_id "MSTRG.8537"; transcript_id "MSTRG.8537.1"; exon_number "15";

scaffold_5 StringTie transcript 1882159 1888972 1000 - . gene_id "MSTRG.8537"; transcript_id "MSTRG.8537.3";

scaffold_5 StringTie exon 1882159 1882173 1000 - . gene_id "MSTRG.8537"; transcript_id "MSTRG.8537.3"; exon_number "1";

scaffold_5 StringTie exon 1883185 1885641 1000 - . gene_id "MSTRG.8537"; transcript_id "MSTRG.8537.3"; exon_number "2";

scaffold_5 StringTie exon 1885710 1885779 1000 - . gene_id "MSTRG.8537"; transcript_id "MSTRG.8537.3"; exon_number "3";

scaffold_5 StringTie exon 1885867 1886015 1000 - . gene_id "MSTRG.8537"; transcript_id "MSTRG.8537.3"; exon_number "4";

scaffold_5 StringTie exon 1886089 1888067 1000 - . gene_id "MSTRG.8537"; transcript_id "MSTRG.8537.3"; exon_number "5";

scaffold_5 StringTie exon 1888139 1888184 1000 - . gene_id "MSTRG.8537"; transcript_id "MSTRG.8537.3"; exon_number "6";

scaffold_5 StringTie exon 1888263 1888359 1000 - . gene_id "MSTRG.8537"; transcript_id "MSTRG.8537.3"; exon_number "7";

scaffold_5 StringTie exon 1888430 1888972 1000 - . gene_id "MSTRG.8537"; transcript_id "MSTRG.8537.3"; exon_number "8";

scaffold_5 StringTie transcript 4119325 4125981 1000 - . gene_id "MSTRG.8727"; transcript_id "PAC4GC:713309"; gene_name "Vocar.0005s0510"; ref_gene_id "Vocar.0005s0510";

scaffold_5 StringTie exon 4119325 4120328 1000 - . gene_id "MSTRG.8727"; transcript_id "PAC4GC:713309"; exon_number "1"; gene_name "Vocar.0005s0510"; ref_gene_id "Vocar.0005s0510";

scaffold_5 StringTie exon 4120846 4120941 1000 - . gene_id "MSTRG.8727"; transcript_id "PAC4GC:713309"; exon_number "2"; gene_name "Vocar.0005s0510"; ref_gene_id "Vocar.0005s0510";

scaffold_5 StringTie exon 4121442 4121516 1000 - . gene_id "MSTRG.8727"; transcript_id "PAC4GC:713309"; exon_number "3"; gene_name "Vocar.0005s0510"; ref_gene_id "Vocar.0005s0510";

scaffold_5 StringTie exon 4121927 4121986 1000 - . gene_id "MSTRG.8727"; transcript_id "PAC4GC:713309"; exon_number "4"; gene_name "Vocar.0005s0510"; ref_gene_id "Vocar.0005s0510";

scaffold_5 StringTie exon 4122239 4122317 1000 - . gene_id "MSTRG.8727"; transcript_id "PAC4GC:713309"; exon_number "5"; gene_name "Vocar.0005s0510"; ref_gene_id "Vocar.0005s0510";

scaffold_5 StringTie exon 4122512 4125981 1000 - . gene_id "MSTRG.8727"; transcript_id "PAC4GC:713309"; exon_number "6"; gene_name "Vocar.0005s0510"; ref_gene_id "Vocar.0005s0510";

scaffold_5 StringTie transcript 4119325 4132221 1000 - . gene_id "MSTRG.8727"; transcript_id "MSTRG.8727.1";

scaffold_5 StringTie exon 4119325 4120328 1000 - . gene_id "MSTRG.8727"; transcript_id "MSTRG.8727.1"; exon_number "1";

scaffold_5 StringTie exon 4120846 4120941 1000 - . gene_id "MSTRG.8727"; transcript_id "MSTRG.8727.1"; exon_number "2";

scaffold_5 StringTie exon 4121442 4121516 1000 - . gene_id "MSTRG.8727"; transcript_id "MSTRG.8727.1"; exon_number "3";

scaffold_5 StringTie exon 4121927 4121986 1000 - . gene_id "MSTRG.8727"; transcript_id "MSTRG.8727.1"; exon_number "4";

scaffold_5 StringTie exon 4122239 4122317 1000 - . gene_id "MSTRG.8727"; transcript_id "MSTRG.8727.1"; exon_number "5";

scaffold_5 StringTie exon 4122512 4126909 1000 - . gene_id "MSTRG.8727"; transcript_id "MSTRG.8727.1"; exon_number "6";

scaffold_5 StringTie exon 4127059 4127170 1000 - . gene_id "MSTRG.8727"; transcript_id "MSTRG.8727.1"; exon_number "7";

scaffold_5 StringTie exon 4127419 4127494 1000 - . gene_id "MSTRG.8727"; transcript_id "MSTRG.8727.1"; exon_number "8";

scaffold_5 StringTie exon 4127779 4128033 1000 - . gene_id "MSTRG.8727"; transcript_id "MSTRG.8727.1"; exon_number "9";

scaffold_5 StringTie exon 4128396 4128459 1000 - . gene_id "MSTRG.8727"; transcript_id "MSTRG.8727.1"; exon_number "10";

scaffold_5 StringTie exon 4128707 4128783 1000 - . gene_id "MSTRG.8727"; transcript_id "MSTRG.8727.1"; exon_number "11";

scaffold_5 StringTie exon 4129201 4129385 1000 - . gene_id "MSTRG.8727"; transcript_id "MSTRG.8727.1"; exon_number "12";

scaffold_5 StringTie exon 4129722 4130448 1000 - . gene_id "MSTRG.8727"; transcript_id "MSTRG.8727.1"; exon_number "13";

scaffold_5 StringTie exon 4131035 4131194 1000 - . gene_id "MSTRG.8727"; transcript_id "MSTRG.8727.1"; exon_number "14";

scaffold_5 StringTie exon 4131472 4131699 1000 - . gene_id "MSTRG.8727"; transcript_id "MSTRG.8727.1"; exon_number "15";

scaffold_5 StringTie exon 4131931 4132221 1000 - . gene_id "MSTRG.8727"; transcript_id "MSTRG.8727.1"; exon_number "16";

scaffold_55 StringTie transcript 124618 128867 1000 - . gene_id "MSTRG.8980"; transcript_id "MSTRG.8980.2";

scaffold_55 StringTie exon 124618 125229 1000 - . gene_id "MSTRG.8980"; transcript_id "MSTRG.8980.2"; exon_number "1";

scaffold_55 StringTie exon 125439 126874 1000 - . gene_id "MSTRG.8980"; transcript_id "MSTRG.8980.2"; exon_number "2";

scaffold_55 StringTie exon 127236 128867 1000 - . gene_id "MSTRG.8980"; transcript_id "MSTRG.8980.2"; exon_number "3";

scaffold_55 StringTie transcript 124618 128881 1000 - . gene_id "MSTRG.8980"; transcript_id "PAC4GC:721171"; gene_name "Vocar.0055s0013"; ref_gene_id "Vocar.0055s0013";

scaffold_55 StringTie exon 124618 126874 1000 - . gene_id "MSTRG.8980"; transcript_id "PAC4GC:721171"; exon_number "1"; gene_name "Vocar.0055s0013"; ref_gene_id "Vocar.0055s0013";

scaffold_55 StringTie exon 127236 128881 1000 - . gene_id "MSTRG.8980"; transcript_id "PAC4GC:721171"; exon_number "2"; gene_name "Vocar.0055s0013"; ref_gene_id "Vocar.0055s0013";

scaffold_9 StringTie transcript 2748631 2758128 1000 + . gene_id "MSTRG.11003"; transcript_id "PAC4GC:723068"; gene_name "Vocar.0009s0279"; ref_gene_id "Vocar.0009s0279";

scaffold_9 StringTie exon 2748631 2749029 1000 + . gene_id "MSTRG.11003"; transcript_id "PAC4GC:723068"; exon_number "1"; gene_name "Vocar.0009s0279"; ref_gene_id "Vocar.0009s0279";

scaffold_9 StringTie exon 2749654 2749792 1000 + . gene_id "MSTRG.11003"; transcript_id "PAC4GC:723068"; exon_number "2"; gene_name "Vocar.0009s0279"; ref_gene_id "Vocar.0009s0279";

scaffold_9 StringTie exon 2750042 2750134 1000 + . gene_id "MSTRG.11003"; transcript_id "PAC4GC:723068"; exon_number "3"; gene_name "Vocar.0009s0279"; ref_gene_id "Vocar.0009s0279";

scaffold_9 StringTie exon 2750729 2750861 1000 + . gene_id "MSTRG.11003"; transcript_id "PAC4GC:723068"; exon_number "4"; gene_name "Vocar.0009s0279"; ref_gene_id "Vocar.0009s0279";

scaffold_9 StringTie exon 2751247 2751399 1000 + . gene_id "MSTRG.11003"; transcript_id "PAC4GC:723068"; exon_number "5"; gene_name "Vocar.0009s0279"; ref_gene_id "Vocar.0009s0279";

scaffold_9 StringTie exon 2751991 2752215 1000 + . gene_id "MSTRG.11003"; transcript_id "PAC4GC:723068"; exon_number "6"; gene_name "Vocar.0009s0279"; ref_gene_id "Vocar.0009s0279";

scaffold_9 StringTie exon 2752655 2752930 1000 + . gene_id "MSTRG.11003"; transcript_id "PAC4GC:723068"; exon_number "7"; gene_name "Vocar.0009s0279"; ref_gene_id "Vocar.0009s0279";

scaffold_9 StringTie exon 2753144 2753324 1000 + . gene_id "MSTRG.11003"; transcript_id "PAC4GC:723068"; exon_number "8"; gene_name "Vocar.0009s0279"; ref_gene_id "Vocar.0009s0279";

scaffold_9 StringTie exon 2753642 2753743 1000 + . gene_id "MSTRG.11003"; transcript_id "PAC4GC:723068"; exon_number "9"; gene_name "Vocar.0009s0279"; ref_gene_id "Vocar.0009s0279";

scaffold_9 StringTie exon 2754331 2754419 1000 + . gene_id "MSTRG.11003"; transcript_id "PAC4GC:723068"; exon_number "10"; gene_name "Vocar.0009s0279"; ref_gene_id "Vocar.0009s0279";

scaffold_9 StringTie exon 2754442 2754533 1000 + . gene_id "MSTRG.11003"; transcript_id "PAC4GC:723068"; exon_number "11"; gene_name "Vocar.0009s0279"; ref_gene_id "Vocar.0009s0279";

scaffold_9 StringTie exon 2754815 2754882 1000 + . gene_id "MSTRG.11003"; transcript_id "PAC4GC:723068"; exon_number "12"; gene_name "Vocar.0009s0279"; ref_gene_id "Vocar.0009s0279";

scaffold_9 StringTie exon 2754907 2754959 1000 + . gene_id "MSTRG.11003"; transcript_id "PAC4GC:723068"; exon_number "13"; gene_name "Vocar.0009s0279"; ref_gene_id "Vocar.0009s0279";

scaffold_9 StringTie exon 2755090 2755341 1000 + . gene_id "MSTRG.11003"; transcript_id "PAC4GC:723068"; exon_number "14"; gene_name "Vocar.0009s0279"; ref_gene_id "Vocar.0009s0279";

scaffold_9 StringTie exon 2755530 2755631 1000 + . gene_id "MSTRG.11003"; transcript_id "PAC4GC:723068"; exon_number "15"; gene_name "Vocar.0009s0279"; ref_gene_id "Vocar.0009s0279";

scaffold_9 StringTie exon 2755744 2755890 1000 + . gene_id "MSTRG.11003"; transcript_id "PAC4GC:723068"; exon_number "16"; gene_name "Vocar.0009s0279"; ref_gene_id "Vocar.0009s0279";

scaffold_9 StringTie exon 2755997 2756061 1000 + . gene_id "MSTRG.11003"; transcript_id "PAC4GC:723068"; exon_number "17"; gene_name "Vocar.0009s0279"; ref_gene_id "Vocar.0009s0279";

scaffold_9 StringTie exon 2756406 2756451 1000 + . gene_id "MSTRG.11003"; transcript_id "PAC4GC:723068"; exon_number "18"; gene_name "Vocar.0009s0279"; ref_gene_id "Vocar.0009s0279";

scaffold_9 StringTie exon 2756766 2757068 1000 + . gene_id "MSTRG.11003"; transcript_id "PAC4GC:723068"; exon_number "19"; gene_name "Vocar.0009s0279"; ref_gene_id "Vocar.0009s0279";

scaffold_9 StringTie exon 2757449 2757559 1000 + . gene_id "MSTRG.11003"; transcript_id "PAC4GC:723068"; exon_number "20"; gene_name "Vocar.0009s0279"; ref_gene_id "Vocar.0009s0279";

scaffold_9 StringTie exon 2757871 2758128 1000 + . gene_id "MSTRG.11003"; transcript_id "PAC4GC:723068"; exon_number "21"; gene_name "Vocar.0009s0279"; ref_gene_id "Vocar.0009s0279";

scaffold_9 StringTie transcript 2754533 2758873 1000 + . gene_id "MSTRG.11003"; transcript_id "MSTRG.11003.2";

scaffold_9 StringTie exon 2754533 2754533 1000 + . gene_id "MSTRG.11003"; transcript_id "MSTRG.11003.2"; exon_number "1";

scaffold_9 StringTie exon 2754815 2754882 1000 + . gene_id "MSTRG.11003"; transcript_id "MSTRG.11003.2"; exon_number "2";

scaffold_9 StringTie exon 2755090 2755341 1000 + . gene_id "MSTRG.11003"; transcript_id "MSTRG.11003.2"; exon_number "3";

scaffold_9 StringTie exon 2755530 2755631 1000 + . gene_id "MSTRG.11003"; transcript_id "MSTRG.11003.2"; exon_number "4";

scaffold_9 StringTie exon 2755997 2756061 1000 + . gene_id "MSTRG.11003"; transcript_id "MSTRG.11003.2"; exon_number "5";

scaffold_9 StringTie exon 2756406 2756451 1000 + . gene_id "MSTRG.11003"; transcript_id "MSTRG.11003.2"; exon_number "6";

scaffold_9 StringTie exon 2756766 2757068 1000 + . gene_id "MSTRG.11003"; transcript_id "MSTRG.11003.2"; exon_number "7";

scaffold_9 StringTie exon 2757449 2757559 1000 + . gene_id "MSTRG.11003"; transcript_id "MSTRG.11003.2"; exon_number "8";

scaffold_9 StringTie exon 2757871 2758873 1000 + . gene_id "MSTRG.11003"; transcript_id "MSTRG.11003.2"; exon_number "9";

scaffold_97 StringTie transcript 840 2813 1000 - . gene_id "MSTRG.11131"; transcript_id "Vocar20014470m.2.0"; gene_name "Vocar20014470m.g"; ref_gene_id "Vocar20014470m.g.2.0";

scaffold_97 StringTie exon 840 2813 1000 - . gene_id "MSTRG.11131"; transcript_id "Vocar20014470m.2.0"; exon_number "1"; gene_name "Vocar20014470m.g"; ref_gene_id "Vocar20014470m.g.2.0";

scaffold_97 StringTie transcript 840 4761 1000 - . gene_id "MSTRG.11131"; transcript_id "MSTRG.11131.1";

scaffold_97 StringTie exon 840 3383 1000 - . gene_id "MSTRG.11131"; transcript_id "MSTRG.11131.1"; exon_number "1";

scaffold_97 StringTie exon 3574 3677 1000 - . gene_id "MSTRG.11131"; transcript_id "MSTRG.11131.1"; exon_number "2";

scaffold_97 StringTie exon 4564 4761 1000 - . gene_id "MSTRG.11131"; transcript_id "MSTRG.11131.1"; exon_number "3";
